# Characterization of interventional clinical trials for monkeypox; systematic review of ClinicalTrials.gov database

**DOI:** 10.3389/fpubh.2023.1144325

**Published:** 2023-03-09

**Authors:** Nasser M. Alorfi, Fahad S. Alshehri, Abdul Haseeb

**Affiliations:** ^1^Department of Pharmacology and Toxicology, College of Pharmacy, Umm Al-Qura University, Makkah, Saudi Arabia; ^2^Department of Clinical Pharmacy, College of Pharmacy, Umm Al-Qura University, Makkah, Saudi Arabia

**Keywords:** monkeypox, viral, clinical trials, vaccination, immunity

## Abstract

**Background:**

Monkeypox (mpox), a zoonotic viral infection, poses a global threat that is being acknowledged at the national and international levels. This systematic review aims to identify and characterize interventional clinical trials for mpox.

**Method:**

All interventional clinical trials registered at ClinicalTrials.gov for mpox were searched up to January 6, 2023. We described the characteristics of interventional clinical trials, and drug interventions (including drugs and vaccines).

**Results:**

As of January 6, 2023, there were 10 clinical trials in the ClinicalTrials.gov registry that met our criteria. Most of the interventional clinical trials were focused on the treatment (*N* = 4, 40%) and prevention (*N* = 4, 40%) of mpox. From the 10 trials, 50% used random treatment allocation, and six (60%) chose the parallel assignment intervention model. All 10 studies were blinded, and six were open-label blinded. The largest proportion of the clinical trials (*N* = 4, 40%) were registered in Europe, followed by America (*N* = 3, 30%) and Africa and others (*N* = 3, 30%). The JYNNEOS vaccine (40%), followed by Tecovirimat (30%) were the most frequently studied drugs used against mpox.

**Conclusion:**

A limited number of clinical trials have been registered on ClinicalTrials.gov since the first case of mpox was reported. Therefore, there is an urgent need to conduct large-scale randomized clinical trials to assess the safety and efficacy of the drugs and vaccines being used against the mpox virus.

## Introduction

In addition to the global scourge of Coronavirus disease 2019 (COVID-19), the monkeypox virus (MPXV) has raised health authorities' concerns (1). Monkeypox (mpox) is a zoonotic viral infection caused by MPXV, a double-stranded DNA virus of the genus orthopox. The smallpox virus, camelpox virus (CMLV), cowpox virus (CPXV), and vaccinia also belong to this genus (2). As early as 1958, mpox was confirmed to contain MPXV, but it was 1970 in Congo until the first case in humans was identified (3, 4). Mpox shares many of the clinical features of smallpox, typically headache, tiredness, rashes, fever and lesions. These lesions evolve sequentially from macules to papules, vesicles and pastules and then crusts, which later on dry up and fall off (5).

The World Health Organization (WHO) has received reports of mpox cases from all around the world. According to preliminary data from the WHO, a total of 84,330 laboratory-confirmed cases and 1,343 suspected cases of mpox had been reported from over 100 countries by January 6, 2023 (6). According to geographic distribution, the regions of America, Africa, Europe, South East Asia, and the Eastern Mediterranean account for most of the confirmed cases of mpox (7, 8). The WHO has reported 74 deaths, emphasizing the importance of conducting further public health investigations in countries where mpox is not endemic. This includes identifying cases, careful contact tracing, maintaining effective surveillance, conducting laboratory tests, and managing clinical care (9).

Global health concerns have been raised by current mpox and COVID-19 outbreaks (10). The WHO authorities have issued interim guidelines for public health officials and healthcare professionals regarding the use of mpox vaccines for the prevention of viral infection (11). Previous foreign studies reported that children are particularly susceptible, as those aged 15 years and under account for 90% of mpox cases (12). Insufficient laboratory diagnostics, vaccines, and antivirals may hinder the effective clinical management of patients with confirmed mpox cases (13). Most researchers are conducting clinical trials to find effective treatments for mpox, evaluate the safety and efficacy of existing antivirals, reduce mortality and morbidity rates, and assess the pharmacokinetic parameters of certain drugs.

ClinicalTrials.gov is the biggest clinical trial database currently available. A previous publication described the organization's process for registration and its use for analyzing clinical trials (14). In this review, we focused on the ongoing clinical trials for mpox registered on ClinicalTrials.gov in order to identify and characterize interventional clinical trials for mpox. We believe this to be important because the incidence rate of mpox is expected to escalate and we need to take necessary precautions, including effective surveillance, isolation, and contact tracing.

## Methods

### Clinical trials search

On January 6, 2023, relevant studies were searched for on ClinicalTrials.gov using the single search term “Monkeypox.” The dataset for the monkeypox clinical trials was limited to only include interventional studies that were registered within the larger data system, up to that date. Any registered clinical trials that had been terminated, withdrawn or suspended were also excluded from the final analysis.

### Clinical trials collection

Two of the reviewers independently collected data from the downloaded registration information files, after which the data were reviewed by a third reviewer. The data collected included the aim of the study, the type of study, the study design, the inclusion criteria, sponsors, estimated enrolment, where the trial was conducted, and other protocol information.

We conducted the analysis, describing the results of interventional clinical trials, and trials on drug interventions, including vaccines and antivirals. Drugs administered, imaging technologies and surgical procedures were categorized as either standard treatment or experimental intervention based on their role in the trial. If a procedure was specifically targeted for a particular trial, it was categorized as an experimental intervention.

We grouped the study locations into continents based on the website allocation. Also, we categorized the funding sources under “sponsors” in the ClinicalTrials.gov database into government funding agency, medical institute or research institute. If a study was not sponsored by any of the aforementioned funding bodies, it was categorized as “others”.

## Results

### Number of studies

**Figure 1:**
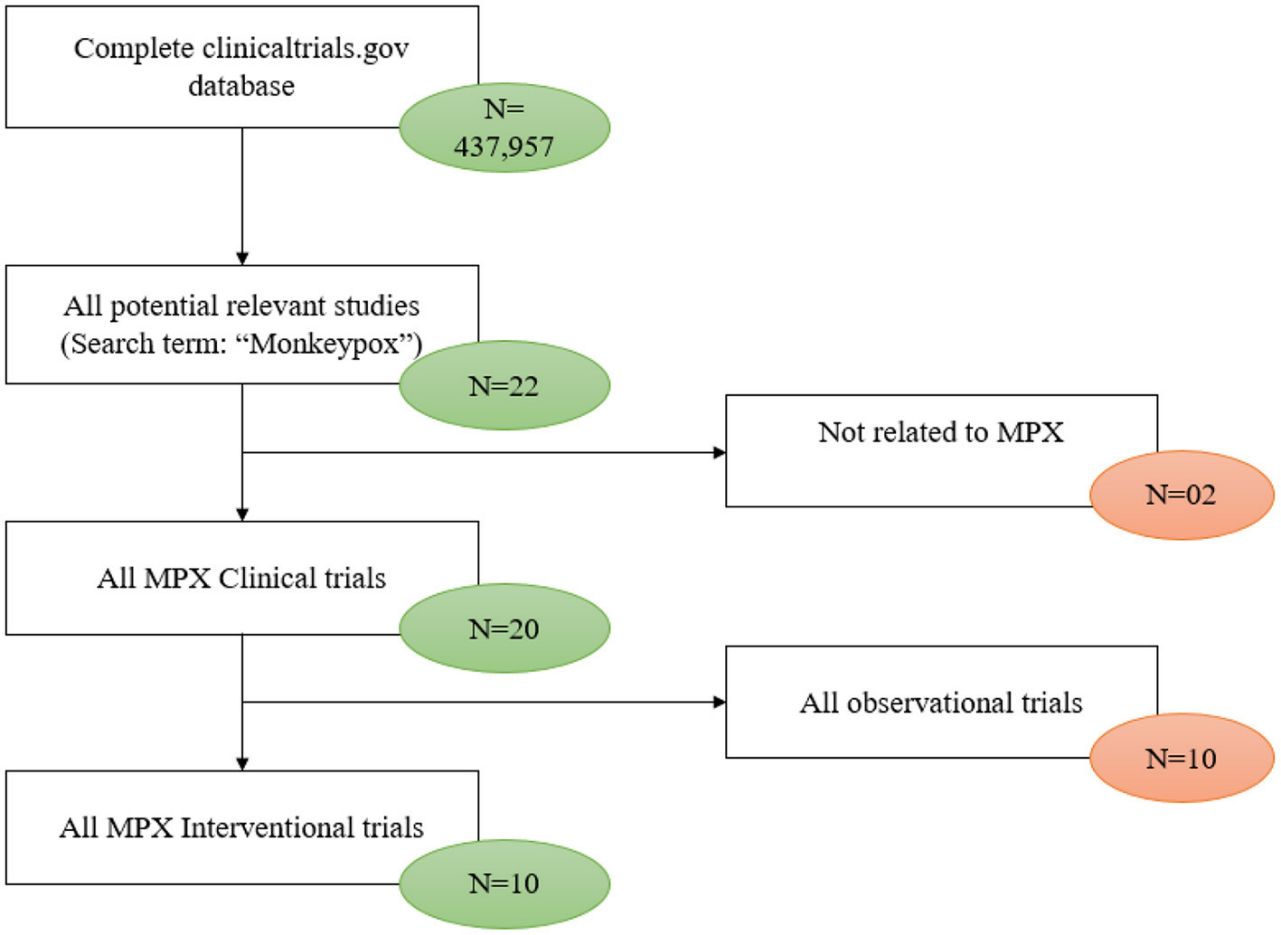
Flow chart of clinical trial selection process.

As of January 6, 2023, a total of 22 registered clinical trials related to mpox were identified in the ClinicalTrials.gov database. Two studies were excluded because they did not provide any information about mpox, and 10 studies were excluded because they were observational studies. The remaining 10 studies were then available to be analyzed (Figure 1).

### Characteristics of interventional trials

**Figure 2:**
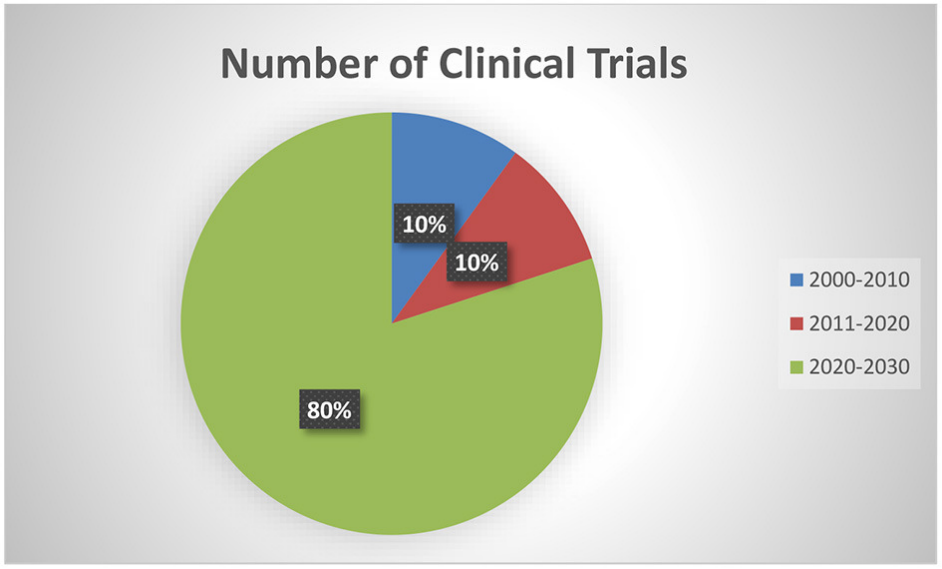
Number of clinical trials conducted in years.

**Figure 3:**
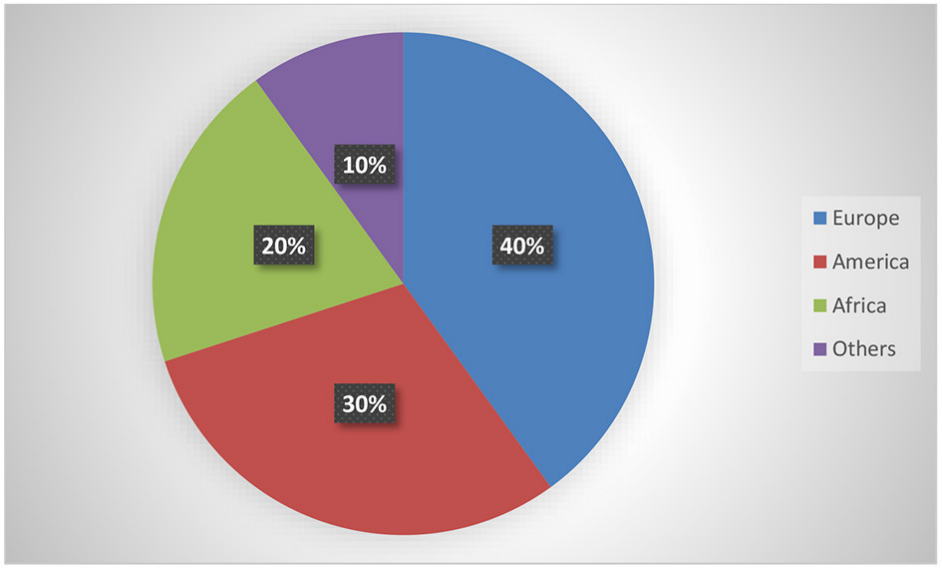
Geographical distribution of clinical trials.

Table 1 depicts the characteristics of the 10 interventional trials. Five (50%) had not started recruiting and four (40%) were still recruiting. Most of the interventional clinical trials were focused on the treatment (*N* = 4, 40%) and prevention (*N* = 4, 40%) of mpox. Treatment allocation was randomized in five clinical trials (50%) and six clinical trials (60%) chose the parallel assignment intervention model. All of the studies were blinded, with six employing open-label blinding. Half had 250 or fewer participants. The majority of the clinical trials were performed with adults aged above 18 years old. Government agencies and medical institutes sponsored 40 and 30% of the trials, respectively. Of these, two projects were funded by a Federal US agency and one project was funded by CDC. Figure 2 illustrates that most of the clinical trials (*N* = 8, 80%) were conducted in 2022. The most common countries for the clinical trials were Europe (*N* = 4, 40%), followed by America (*N* = 3, 30.00%) and Africa (*N* = 2, 20%) as shown in Figure 3.

**Table 1:** Characteristics of interventional clinical trials registered on ClinicalTrials.gov.

	**Number (*N*)**	**Percentage (%)**
**Interventional model**		
Single group assignment	4	40.00
Parallel assignment	6	60.00
**Recruitment status**		
Recruiting	4	40.00
Active, not recruiting	1	10.00
Not yet recruiting	5	50.00
**Study purpose**		
Prevention	4	40.00
Treatment	4	40.00
Supportive care	1	10.00
Others	1	10.00
**Sample size**		
0–250	5	50.00
251–500	2	20.00
>500	2	20.00
Not available	1	10.00
**Trial phase**		
Phase 2	2	20.00
Phase 3	4	40.00
Not available	4	40.00
**Treatment allocation**		
Randomized	5	50.00
Non-randomized	1	10.00
NR	4	40.00
**Masking (blinding)**		
Double	2	20.00
Open	6	60.00
Others	2	20.00
**Funding source**		
Government	4	40.00
Medical institution	3	40.00
Others	3	30.00

### Analysis of drugs studied in clinical trials

As shown in Table 2, tecovirimat was used for the treatment of patients with confirmed mpox while the vaccine Imvanex (also known as JYNNEOS and Imvamune) was used for the prevention of mpox.

**Table 2:** Most studied drugs in clinical trials.

**Drugs/vaccines**	**Number of trials**
Tecovirimat/ST-246	4
Imvanex/JYNNEOS/Imvamune	3
Others	3

#### Tecovirimat

Patients were treated with tecovirimat (also named ST-246) or a placebo for 14 days, each being administered in a hospital with standard-of-care (SOC) treatment. Afterwards, the patients were examined weekly for 28 days to further evaluate their mpox infection, and to carry out a safety assessment. Participants were then followed-up with an optional visit between days 57 and 59 for possible recrudescence of the infection.

In a double-blind, randomized, cross-over study of the pharmacokinetics of an oral dose of anti-orthopoxvirus compound, a single dose of ST-246 was administered in healthy patients with mpox. The primary outcome measures were the assessment of the pharmacokinetic parameters of a single dose of ST-246. Six patients received ST-246 Form V (hemihydrate) followed 10 days later after a wash-out period by Form I (monohydrate), and the remaining received ST-246 Form I (monohydrate) followed by Form V (hemihydrate).

#### JYNNEOS/Imvamune/Imvanex

Healthy volunteers aged 18 years or older participated in the open-label clinical trial. Participants receive the vaccine Imvamune on days 0 and 28. Blood samples were collected on days 0, 14, 28, 42, 280, 365, 545, and 730 for immunogenicity analysis. The participants were observed for at least 30 min to assess if any adverse events occurred. The recorded exposure to mpox was also maintained at each follow-up visit.

## Discussion

This systematic review provides an initial overview of the mpox clinical trials that have been registered on ClinicalTrials.gov, with a specific emphasis on treating and managing mpox. Multiple noteworthy observations emerged from this review of interventional clinical trials. We found a limited number of mpox clinical trials. Most of the interventional trials enrolled <250 participants.

Small-scale studies are prone to type II errors, otherwise known as a false negative, which occur when the null hypothesis is not rejected. This leads to the inaccurate conclusion that an intervention or treatment is ineffective, because the sample is of an inadequate size, meaning that it is difficult to detect a significant effect (15). Although many interventional clinical trials share favorable features in their design, such as similar rates of randomized and non-randomized trials with two treatment arms across different time periods, some clinical trials also exhibit unfavorable study design characteristics, such as those with active comparator data monitoring committees (as observed in clinical trials NCT05597735 and NCT05534165). These findings underscore the need for better-designed trials and monitoring practices.

Mpox management varies from patient to patient and should involve decision making centered on patients, and multidisciplinary discussions that aim to augment disease control, reduce and improving quality of life among patients (16). Current treatment options for patients with mpox infection include vaccines such as JYNNEOS and administration of an antiviral drug, i.e., tecovirimat (17). Tecovirimat is a FDA-approved drug that is used for the treatment of smallpox. Prior to the onset of mpox, a randomized clinical trial (RCT) was being planned by the National Institute of Health (NIH) in Congo with the aim of evaluating the effectiveness and safety of tecovirimat (18). Based on a study with healthy volunteers, tecovirimat appears to have a favorable clinical profile (19). A double-blind, randomized, cross-over clinical trial for the assessment of the anti-orthopoxvirus compound, ST-246, is in process to assess the pharmacokinetic parameters of an oral dose of that drug in patients with mpox. Pharmacometrics utilized pharmacokinetic and pharmacodynamic data to establish models that describe drug efficacy factors such as the progression of disease, compliance with treatment, and viral growth. These models provide guidance for the design of trials, comparisons of effectiveness, changes in drug dosage, and decision making for patient care in particular populations (20).

Our analysis shows that the four clinical trials focused on the prevention and management of mpox. Previous studies have reported that smallpox vaccines could provide some protection against mpox and lessen its clinical manifestations (5, 21, 22). Currently, JYNNEOS (also named as Imvanex, MVA-BN, or Imvamune) is approved for smallpox and it is being studies as a potential drug that can prevent infections with MPXV (23). JYNNEOS is a viral vaccine made from the MVA-BN strain, which is a modified vaccinia Ankara-Bavarian Nordic strain of orthopoxvirus that has been weakened and is no longer able to reproduce itself (24). This vaccine was licensed by US-FDA on September 24, 2019 and is currently recommended as a vaccine for mpox and small pox, for adults (>18 years old) who are classified as being at high risk of exposure to these viral diseases (25). In most of the interventional clinical trials, the Immavune vaccine was given to healthy volunteers on days 0 and 28 for the prevention of MPXV.

The current mpox outbreak requires a multi-faceted approach that includes more than just treatment (26). Public awareness, robust testing, containment measures and vaccination of high-risk groups all have significant roles to play in minimizing the spread of MPXV (27, 28). Nonetheless, significant challenges persist. While vaccines are believed to be safe and effective for individuals with smallpox infection, there is a shortage of data on their effectiveness in terms of managing mpox. The re-emergence of this zoonotic viral disease raises concerns, and additional research is merited into measures and treatments that could be employed to help prevent and combat the disease, which is now present in multiple nations through possible new routes of transmission. To the best of our understanding, key interventions to prevent mpox outbreaks include early identification, barrier nursing, raising awareness, and strict infection prevention control practices that include isolating individuals diagnosed with mpox. This requires efforts by public health officials and healthcare professionals. Also, epidemiological studies should focus on how the virus can be transmitted from animals to people and identify possible sources of infections, such as people who work closely with animals (29, 30). Furthermore, more clinical trials should be carried out to ensure that patients receive optimal care.

## Conclusion

The periodic occurrence and frequency of mpox outbreaks bring to light the need for outbreak awareness, research and preparedness. There is only a small number of interventional clinical trials registered on ClinicalTrials.gov that are focusing on mpox vaccines (JYNNEOS) and antiviral drugs (tecovirimat), and assessing how effective and safe they are. Therefore, we call for more large-scale randomized clinical trials to be conducted in order to assess mpox drugs and vaccines, particularly in Africa.

## Data availability statement

The original contributions presented in the study are included in the article/supplementary material, further inquiries can be directed to the corresponding author.

## Author contributions

All authors wrote sections of the manuscript, contributed to manuscript revision, read, and approved the submitted version.

## References

[1] Khan S Akbar SMF Yahiro T Mahtab MA Kimitsuki K Nishizono A. Unprecedented rise of monkeypox in Europe and America: Are Asian countries ready for a new outbreak during the ongoing COVID-19 pandemic? J Glob Health. (2022) 12:e03066. 10.7189/jogh.12.03066 36039839 PMC9425425

[2] Sepehrinezhad A Ashayeri Ahmadabad R Sahab-Negah S. Monkeypox virus from neurological complications to neuroinvasive properties: Current status and future perspectives. J Neurol. (2023) 270:101–8. 10.1007/s00415-022-11339-w 35989372 PMC9393054

[3] Hraib M Jouni S Albitar MM Alaidi S Alshehabi Z. The outbreak of monkeypox 2022: An overview. Ann Med Surg. (2022) 79:104069. 10.1016/j.amsu.2022.104069 35860140 PMC9289401

[4] Kmiec D Kirchhoff F. Monkeypox: A new threat? Int J Mol Sci. (2022) 23:7866. 10.3390/ijms23147866 35887214 PMC9321130

[5] Sudarmaji N Kifli N Hermansyah A Yeoh SF Goh B-H Ming LC. Prevention and treatment of monkeypox: A systematic review of preclinical studies. Viruses. (2022) 14:112496. 10.3390/v14112496 36423105 PMC9699130

[6] WHO. 2022 Mpox (Monkeypox) Outbreak: Global Trends. Geneva: WHO (2023).

[7] Taylor L. Monkeypox: WHO to rename disease to prevent stigma. Br Med J. (2022) 2022:bmj.o1489. 10.1136/bmj.o1489 35710105

[8] Abu-Hammad O Abu-Hammad A Jaber A-R Jaber AR Dar-Odeh N. Factors associated with geographic variations in the 2022 monkeypox outbreak; A systematic review. New Microbes New Infect. (2023) 51:101078. 10.1016/j.nmni.2022.101078 36618975 PMC9810380

[9] Awan MAE Waseem M Sahito AF Sahito AM Khatri G Butt MA . Monkeypox has devastated the world; should we prepare for the outbreak of a new pandemic? Ann Med Surg. (2022) 79:104051. 10.1016/j.amsu.2022.104051 35860122 PMC9289421

[10] Saied AA Priyanka Metwally AA Choudhary OP. Monkeypox: An extra burden on global health. Int J Surg. (2022) 104:106745. 10.1016/j.ijsu.2022.106745 35777695 PMC9238059

[11] Petersen E Zumla A Hui DS Blumberg L Valdoleiros SR Amao L . Vaccination for monkeypox prevention in persons with high-risk sexual behaviours to control on-going outbreak of monkeypox virus clade 3. Int J Infect Dis. (2022) 122:569–71. 10.1016/j.ijid.2022.06.047 35788415 PMC9534076

[12] Jiang RM Zheng YJ Zhou L Feng LZ Ma L Xu BP . Diagnosis, treatment, and prevention of monkeypox in children: An experts' consensus statement. World J Pediatr. (2022) 2022:1–12. 10.1007/s12519-022-00624-3 36409451 PMC9685019

[13] Zaheer AB Ali T Ashfaq A Jabeen A. Monkeypox outbreak amidst COVID-19 reemergence in the European Region: Challenges, efforts, and recommendations. Ann Med Surg. (2022) 82:104657. 10.1016/j.amsu.2022.104657 36128260 PMC9479377

[14] Gresham G Meinert JL Gresham AG Piantadosi S Meinert CL. Update on the clinical trial landscape: analysis of ClinicalTrials. gov registration data, 2000–2020. Trials. (2022) 23:1–15. 10.21203/rs.3.rs-1164646/v1 36203212 PMC9540299

[15] Banerjee A Chitnis UB Jadhav SL Bhawalkar JS Chaudhury S. Hypothesis testing, type I and type II errors. Ind Psychiatry J. (2009) 18:127–31. 10.4103/0972-6748.62274 21180491 PMC2996198

[16] Sharma V Aggarwal D Sharma AK. An overview on monkeypox, current paradigms and advances in its vaccination, treatment and clinical management: Trends, scope, promise and challenges. J Pure Appl Microbiol. 16(Suppl.1):3000–12. 10.22207/JPAM.16.SPL1.21

[17] Webb E Rigby I Michelen M Dagens A Cheng V Rojek AM . Availability, scope and quality of monkeypox clinical management guidelines globally: A systematic review. Br Med J Glob Health. (2022) 7:e009838. 10.1136/bmjgh-2022-009838 35973747 PMC9472169

[18] Sherwat A Brooks JT Birnkrant D Kim P. Tecovirimat and the treatment of monkeypox—past, present, and future considerations. N Engl J Med. (2022) 387:579–81. 10.1056/NEJMp2210125 35921403

[19] Desai AN 3rd GRT Neumeister SM Arutyunova AM Trigg K Cohen SH. Compassionate use of tecovirimat for the treatment of monkeypox infection. J Am Med Assoc. (2022) 328:1348–50. 10.1001/jama.2022.15336 35994281 PMC9396467

[20] Palmer ME Andrews LJ Abbey TC Dahlquist AE Wenzler E. The importance of pharmacokinetics and pharmacodynamics in antimicrobial drug development and their influence on the success of agents developed to combat resistant gram negative pathogens: A review. Front Pharmacol. (2022) 13:888079. 10.3389/fphar.2022.888079 35959440 PMC9359604

[21] Chakraborty C Bhattacharya M Sharma AR Dhama K. Monkeypox virus vaccine evolution and global preparedness for vaccination. Int Immunopharmacol. (2022) 113:109346. 10.1016/j.intimp.2022.109346 36274490 PMC9582788

[22] Hemati S Farhadkhani M Sanami S Mohammadi-Moghadam F. A review on insights and lessons from COVID-19 to the prevent of monkeypox pandemic. Travel Med Infect Dis. (2022) 50:102441. 10.1016/j.tmaid.2022.102441 36084881 PMC9446553

[23] Prevention CfDCa. Vaccines. (2022). 23. Available online at: https://www.fda.gov/news-events/press-announcements/fda-approves-first-live-non-replicating-vaccine-prevent-smallpox-and-monkeypox

[24] Rizk JG Lippi G Henry BM Forthal DN Rizk Y. Prevention and treatment of monkeypox. Drugs. (2022) 82:957–63. 35763248 10.1007/s40265-022-01742-y PMC9244487

[25] Rizk JG Lippi G Henry BM Forthal DN Rizk Y. Prevention and treatment of monkeypox. Drugs. (2022) 2022:1–7. 10.1080/14787210.2022.2113058 35763248 PMC9244487

[26] McCarthy MW. Therapeutic strategies to address monkeypox. Expert Rev Anti Infect Ther. (2022) 20:1249–52. 10.1080/14787210.2022.2113058 35953443 PMC9491133

[27] Quarleri J Delpino MV Galvan V. Monkeypox: Considerations for the understanding and containment of the current outbreak in non-endemic countries. Geroscience. (2022) 44:2095–103. 10.1007/s11357-022-00611-6 35726117 PMC9208705

[28] Mahase E. Monkeypox: Healthcare workers will be offered smallpox vaccine as UK buys 20 000 doses. Br Med J. (2022) 377:o1379. 10.1136/bmj.o1379 35649575

[29] Doty JB Malekani JM Kalemba LN Stanley WT Monroe BP Nakazawa YU . Assessing monkeypox virus prevalence in small mammals at the human–animal interface in the Democratic Republic of the Congo. Viruses. (2017) 9:283. 10.3390/v9100283 28972544 PMC5691634

[30] McCollum AM. Epidemiology of human Mpox—Worldwide, 2018–2021. Morbid Mortal Weekly Rep. (2023) 72:3a4. 10.15585/mmwr.mm7203a4 36656790 PMC9869741

